# Modelling of Waning of Immunity and Reinfection Induced Antibody Boosting of SARS-CoV-2 in Manaus, Brazil

**DOI:** 10.3390/ijerph19031729

**Published:** 2022-02-02

**Authors:** Haozhen Wei, Salihu S. Musa, Yanji Zhao, Daihai He

**Affiliations:** Department of Applied Mathematics, The Hong Kong Polytechnic University, Hong Kong, China; hao-zhen.wei@connect.polyu.hk (H.W.); salihu-sabiu.musa@connect.polyu.hk (S.S.M.); yan-ji.zhao@connect.polyu.hk (Y.Z.)

**Keywords:** COVID-19, waning of immunity, boosting of immunity, reinfection

## Abstract

It was reported that the Brazilian city, Manaus, likely exceeded the herd immunity threshold (presumably 60–70%) in November 2020 after the first wave of COVID-19, based on the serological data of a routine blood donor. However, a second wave started in November 2020, when an even higher magnitude of deaths hit the city. The arrival of the second wave coincided with the emergence of the Gamma (P.1) variant of SARS-CoV-2, with higher transmissibility, a younger age profile of cases, and a higher hospitalization rate. Prete et al. (2020 MedRxiv 21256644) found that 8 to 33 of 238 (3.4–13.9%) repeated blood donors likely were infected twice in Manaus between March 2020 and March 2021. It is unclear how this finding can be used to explain the second wave. We propose a simple model which allows reinfection to explain the two-wave pattern in Manaus. We find that the two waves with 30% and 40% infection attack rates, respectively, and a reinfection ratio at 3.4–13.9%, can explain the two waves well. We argue that the second wave was likely because the city had not exceeded the herd immunity level after the first wave. The reinfection likely played a weak role in causing the two waves.

## 1. Introduction

The coronavirus disease 2019 (COVID-19) pandemic has had a tremendous impact globally. Effective vaccines have given us hope to end the pandemic. Several variants of concern (VOCs) of the severe acute respiratory virus 2 (SARS-CoV-2), the virus of COVID-19, emerged with increased transmissibility and immunity-escaping ability and posed new threats [1,2,3,4,5]. The SARS-CoV-2 P.1 variant that likely emerged from Amazonas, Brazil, was first detected by the Japanese National Institute of Infectious Diseases on 6 January 2021 from a returning traveller from Brazil [6]. As of June 2021, the P.1 variant (or Gamma variant) has spread to 55 countries globally and was once the dominant lineage in the South American region. The Amazonas province of Brazil was hit badly by COVID-19. Buss et al. [7] reported that the infection attack rate (IAR) in Manaus reached 76% by November 2020, based on routine blood donor data; thus, an immediate second wave was impossible based on the herd immunity theory. However, an even higher second wave of the P.1 variant hit the city. The 76% IAR of the first wave and a comparable IAR of the second wave (let us assume 76% as well) led to a reinfection ratio, i.e., the proportion of the population being infected twice in a year reached a level of 52% (76–24)%. Namely, if the second wave had been as high as the first wave, every inhabitant in Manaus would have been infected 1.52 times. These numbers are implausible since only three confirmed reinfections were reported in the whole Amazonas province, including Manaus [8]. Prete et al. [9] reported a reinfection ratio at 3–13.9% based on serological antibody level (IgG) data from 238 repeated blood donors. Their finding motivated us to formulate a model to examine the possible role of reinfection in causing the two waves in Manaus.

To the best of our knowledge, there is no clear evidence that the P.1 variant resulted in greater or lesser severity of COVID-19 than pre-existing variants [10]. Nevertheless, the increase in transmissibility led to a significant increase in incidences locally [11].

**Figure 1:**
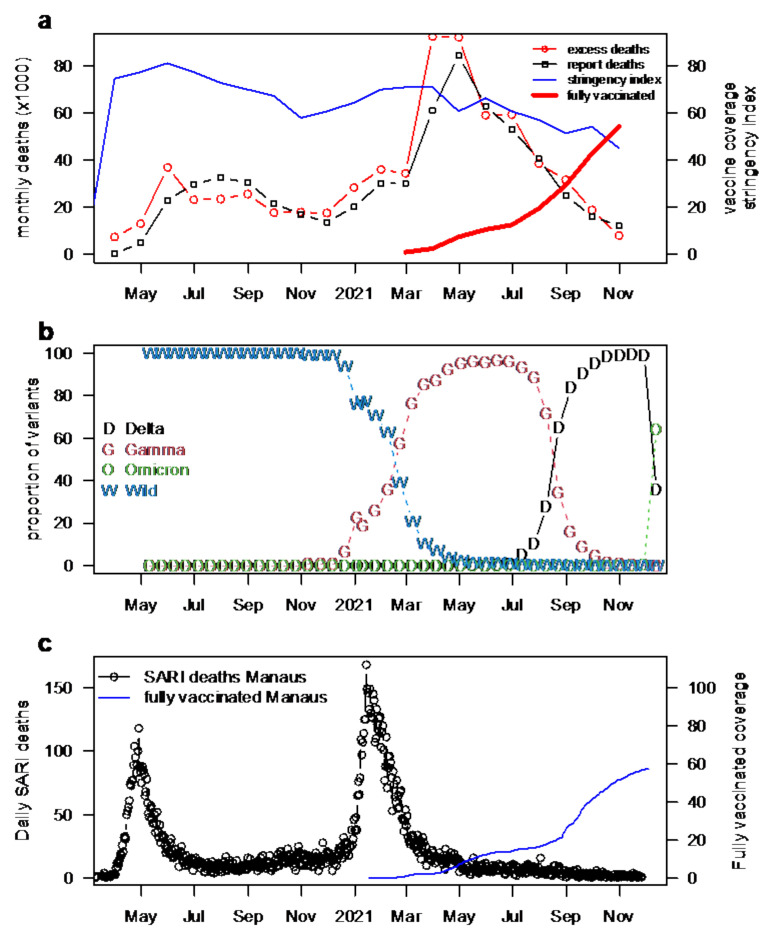
COVID-19 deaths, stringency index and fully vaccinated coverage in Manaus and in Brazil. (**a**) Monthly excess deaths, reported deaths, stringency index and fully vaccinated coverage in Brazil (national level). (**b**) The proportion of variants in Brazil. (**c**) Daily SARI deaths and fully vaccinated coverage in Manaus (city-level). The Gamma (P.1) variant replaced the wild strain and was replaced by the Delta variant. The Omicron variant is replacing the Delta variant. These replacements suggested that the transmissibility advantage of the new variants was higher than their proceeding variants.

Figure 1 shows the COVID-19 pandemic in Brazil. Panel (a) shows the monthly excess deaths (all-cause deaths in pandemic years minus the average level of all-cause of deaths in the previous five years), reported COVID-19 deaths, stringency index (an indicator of government control measure), and the fully vaccinated percentage of the population in Brazil (national level). Panel (b) shows the biweekly variant proportions among all samples sequenced (data from “The our world in data” [12] and GISAID [13,14,15]). Gamma variant replaced the previous wild strain and was replaced by the Delta variant. Now, the Omicron variant is replacing the Delta variant. Panel (c) shows that the city level reported daily severe acute respiratory infection (SARI) deaths with the daily fully vaccinated coverage in Manaus (city-level). The excess deaths matched the reported COVID-19, suggesting that the reporting ratio of COVID-19 deaths is high in Brazil at the population level. The number of SARI deaths is a good proxy of COVID-19 since, during the pandemic, the transmission of other types of SARI was virtually absent. The SARI deaths show two-wave patterns with a higher peak for the second wave in Manaus. We discussed the effect of the vaccination in previous work [16]. In this work, we focus on the period before May 2021, i.e., before the second dose coverage exceeded 10%.

## 2. Methods

We employed a simple conceptual model to examine the impact of reinfection, transmissibility, and severity associated with the increasing proportions of infections with the P.1 variant in Manaus, Brazil. Each reinfection case has a prior infection. We assume that these two infections (reinfection and prior infection) are from two waves, respectively. To quantify the reinfection ratio, one could define (1) the total number of reinfections out of the total number of infections in the first wave; (2) total number of reinfections out of the total number of infections in the second wave; (3) total number of reinfections out of total infections; (4) total number of reinfections out of the total population, in a given time interval. We choose the definition (4) to refer to the reinfection ratio in this work. Two waves of COVID-19 hit Manaus badly (see Figure 1), with more than 13,000 SARI deaths by 11 October 2021, in a city of 2 million.

Prete et al. [9] studied the antibody level (IgG) of 238 repeated blood donors in Manaus, and found that some of them showed evidence of being infected with a wild strain in the first wave, i.e., an increase in their IgG level in April–May 2020. Others showed evidence of being infected with the P.1 strain in the second wave, i.e., elevated IgG levels from December 2020 to January 2021. A proportion of those infected in the first wave showed an immunity decay and boosting pattern (two peaks in their IgG level in both waves) and thus was likely re-infected in the second wave with the P.1 strain. It is a question of whether this pattern can be generalized to the general population. Nevertheless, this finding motivated us to develop a simple conceptual model to describe this phenomenon.

We propose a model (below) to explain the two epidemic waves in Manaus, considering immunity (related to IgG level) decay and boosting. First, we assume that these donors are a good representation of the general population; then, we discuss this assumption later (they are probably not representative). Our model reads:

(1) $$S^{\prime }=-\beta S\left(I+\varepsilon I_{1}\right)$$

(2) $$E^{\prime }=\beta S\left(I+\varepsilon I_{1}\right)-\sigma E$$

(3) $$I^{\prime }=\sigma E-\gamma I$$

(4) $$R^{\prime }=\gamma I-\lambda R$$

(5) $$S_{1}{}^{\prime }=\lambda R-\beta S_{1}\left(I+\varepsilon I_{1}\right)$$

(6) $$E_{1}{}^{\prime }=\beta S_{1}\left(I+\varepsilon I_{1}\right)-\sigma E_{1}$$

(7) $$I_{1}{}^{\prime }=\sigma E_{1}-\gamma I_{1}$$

(8) $$R_{1}{}^{\prime }=\gamma I_{1}$$

The transmission route of susceptible–exposed–infectious–recovered (S-E-I-R) forms the process of primary infection, while the transmission route of S_1_-E_1_-I_1_-R_1_ forms the process of the reinfection. The reinfected individuals could be infectious, with reduced infectiousness which is controlled by a parameter, ε. A set of parameter values were selected such that we can qualitatively reproduce the two wave patterns which occurred in Manaus and the observed reinfection ratio reported by Prete et al. [9].

Parameters β, σ, γ, and λ denote the transmission rate, the rate at which exposed cases move to the infectious class, the rate at which infectious cases move to recovered class, and the rate at which recovered cases lose immunity protection (due to the waning of immunity or the emergence of a variant with immunity-escaping ability). We set $\gamma^{-1}=$ 3 days and $\sigma^{-1}=2\;\mathrm{days}$, such that the sum of the mean latent period and mean infectious period equals five days [17]. First, we consider ε=0; namely, the reinfected cases had no infectiousness, then we show the effects of increasing ε.

We also consider a simpler version of our model, where we set $\gamma^{-1}+\sigma^{-1}\to \gamma^{-1}$, such that

(9) $$S^{\prime }=-\beta S\left(I+\varepsilon I_{1}\right)$$

(10) $$I^{\prime }=\beta S\left(I+\varepsilon I_{1}\right)-\gamma I$$

(11) $$R^{\prime }=\gamma I-\lambda R$$

(12) $$S_{1}{}^{\prime }=\lambda R-\beta S_{1}\left(I+\varepsilon I_{1}\right)$$

(13) $$I_{1}{}^{\prime }=\beta S_{1}\left(I+\varepsilon I_{1}\right)-\gamma I_{1}$$

(14) $$R_{1}{}^{\prime }=\gamma I_{1}$$

At the disease-free equilibrium, the basic reproduction number computed as using the next generation matrix is given by $R_{0}=\frac{\beta }{\gamma }\left(S\left(0\right)+\varepsilon S_{1}\left(0\right)\right)$, where $S\left(0\right)$ and $S_{1}\left(0\right)$ are the initial population of susceptible humans. Given the short generation time, the simpler model without exposed class is a good approximation of the original model [18].

## 3. Results

**Figure 2:**
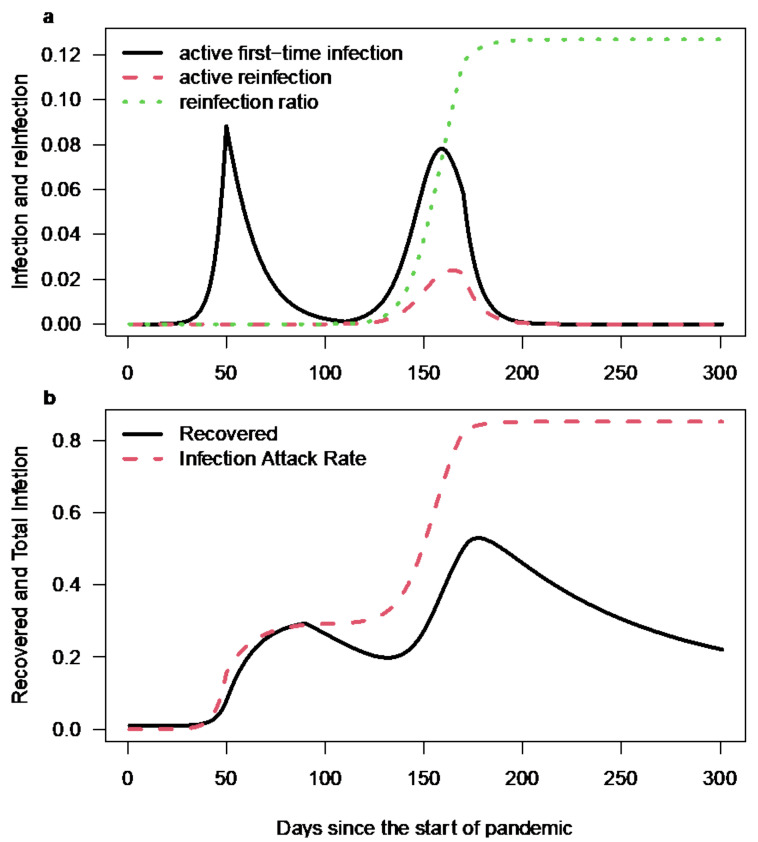
Simulation results of the two waves in Manaus, Brazil, with our model and the Euler integration method. (**a**) The active first-time infection in I class (black curve) and re-infection in I_1_ class (red dashed curve), the reinfection ratio (dotted green curve). (**b**) The recovered and fully protected proportion (black curve) and the infection attack rate (red dashed curve).

Figure 2 shows the simulation results. The parameter β is set as a step function with a high value at $R_{0}=2.5$ (from day 1 to day 49, and from day 109 to day 169) and a low value at $R_{0}=0.8$ (from day 50 to day 110 and after day 169). We set the decay rate λ to be zero in the initial 90 days and 1/80 per day after the initial 90 days to echo the emergence of the P.1 variant; namely, we only allow reinfection due to the P.1 variant. The two waves qualitatively reproduced the two-wave pattern observed in Figure 1. Note that by adding these two curves (black and red), the second wave will have a higher peak than the first. The simulation results in panel (a) of our model reasonably matched the observed two-wave pattern in Figure 1; particularly, the second wave had a higher peak, with a proportion of reinfection at 12.7%, namely 12.7% of the whole population being infected twice. This is in line with Prete et al.’s 3–13.9%. Panel (b) shows the infection attack rate (proportion of the population being infected) and recovered from both first-time infection and infection.

The observed reinfection ratio is about 12.7%, or we may call it the immunity-boosted proportion since we assume that their infectiousness is low. The one recovered from the first wave is only temporarily immunized since we allow immunity decay at a rate λ for those recovered from the first wave. This waning of immunity is partly due to the emergence of the P.1 variant.

**Figure 3:**
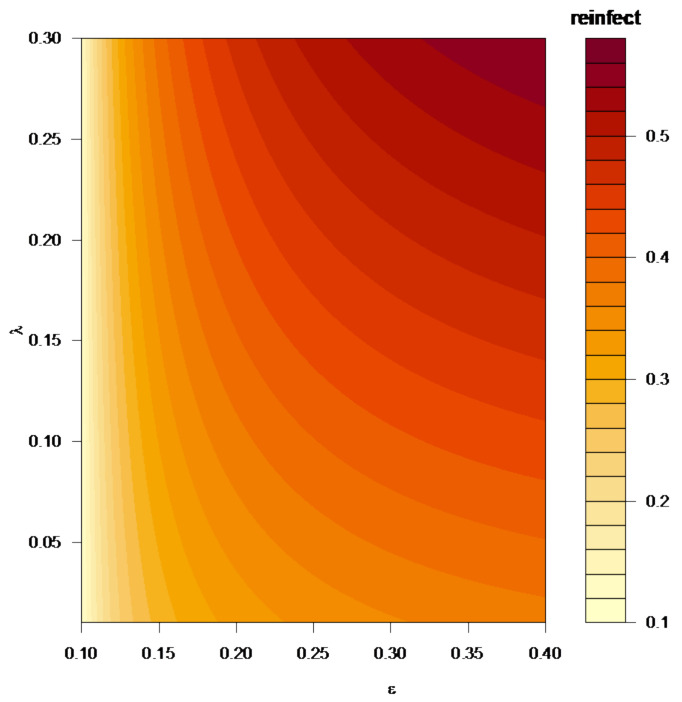
The impact of the decay rate λ and the infectivity of reinfection ε on the reinfection ratio.

Figure 3 shows the impact of λ and ε on the reinfection ratio. We can see that high λ and/or high ε lead to higher reinfection. The rate of reinfection is small when ε is small, regardless of whether the λ is increasing or decreasing; however, when the λ is small with increasing ε, the rate of reinfection is growing. Overall, with the increasing of λ and ε, the color of the reinfection ratio is deeper, which means that the rate of reinfection is larger.

## 4. Discussion

Prete et al.’s work was based on 238 repeated blood donors, who are likely to be socially active and healthy. Thus, whether the finding can be generalized to 2 million people is in question. However, their finding is intriguing, and we wonder whether these immunity-boosted cases were infectious at all and of the same severity as the first-time infection. Before these questions were answered, a rigorous model fitting to their data is not urgent. We may explicitly model the decay of immunity induced by infection with a previous strain as a function of the proportion of P.1. variant. By now, the P1. variant has been replaced by an even more transmissible Delta variant in Brazil. The Omicron variant is replacing the Delta variant. Recent studies suggest that vaccine breakthrough infection and reinfection are more common with the Omicron variant than the Delta and P.1 variants. The key question is the transmissibility and severity of these breakthrough infections and reinfections.

## 5. Conclusions

The mathematical models of infectious diseases have been playing a pivotal role in understanding emerging and re-emerging infectious disease outbreaks, evaluating strategies for effective prevention and control measures, as well as providing suggestions to policymakers on how to effectively control the epidemics in a timely fashion. In this paper, we develop a simple conceptual model to qualitatively reproduce the observed dynamics of SARS-CoV-2 in Manaus with reinfection [9]. The model was able to capture the observed dynamics on reinfection by a new variant [9,19,20,21]. The key message is that this level of reinfection is not against a scenario of an IAR at 30% for the first wave and an IAR at 40% for the second wave. Thus, herd immunity was not reached after the first wave but largely met after the second wave. The reinfection is not a crucial factor in causing the second wave. The second wave was due to low IAR after the first wave; these arguments were supported by independent serological studies [22,23].

Our time-varying transmission rate could be associated with the on and off of control measures in the city and the emergence of the P.1 variant with increased transmissibility. According to a recent study, limited cross-protection between SARS-CoV-2 variants would likely give rise to a higher reinfection ratio [11]. However, some recent studies suggested that SARS-CoV-2 variants conferring increased transmissibility are likely linked to reduced disease severity [11,24]. Existing control and prevention measures seem to be less effective with regard to the new variants; hence, there is a need to have more robust proactive interventions to achieve the same level of control as with the wild strain. In this work, we used a simple conceptual model that assumes homogeneous mixing of population. Although this is not true in reality, e.g., the transmission in urban slums [25] and other regions are very different, and the infection attack rates could thus be very different. More studies should be focused on these disparities.

## Data Availability

https://ourworldindata.org/grapher/covid-variants-area (accessed on 31 December 2021).
